# A missense mutation in *PMEL17 *is associated with the Silver coat color in the horse

**DOI:** 10.1186/1471-2156-7-46

**Published:** 2006-10-09

**Authors:** Emma Brunberg, Leif Andersson, Gus Cothran, Kaj Sandberg, Sofia Mikko, Gabriella Lindgren

**Affiliations:** 1 Dept of Medical Biochemistry and Microbiology, Uppsala University, SE-751 24 Uppsala, Sweden; 2 Dept of Animal Breeding and Genetics, Swedish University of Agricultural Sciences, SE-750 07 Uppsala, Sweden; 3 Dept of Veterinary Science, University of Kentucky, Lexington, KY 40546-0076, USA; 4 Dept of Veterinary Integrative Biosciences, Texas A&M University, College Station, TX 77843-4458, USA

## Abstract

**Background:**

The Silver coat color, also called Silver dapple, in the horse is characterized by dilution of the black pigment in the hair. This phenotype shows an autosomal dominant inheritance. The effect of the mutation is most visible in the long hairs of the mane and tail, which are diluted to a mixture of white and gray hairs. Herein we describe the identification of the responsible gene and a missense mutation associated with the Silver phenotype.

**Results:**

Segregation data on the *Silver *locus (Z) were obtained within one half-sib family that consisted of a heterozygous Silver colored stallion with 34 offspring and their 29 non-Silver dams. We typed 41 genetic markers well spread over the horse genome, including one single microsatellite marker (TKY284) close to the candidate gene *PMEL17 *on horse chromosome 6 (ECA6q23). Significant linkage was found between the Silver phenotype and TKY284 (θ = 0, z = 9.0). DNA sequencing of *PMEL17 *in Silver and non-Silver horses revealed a missense mutation in exon 11 changing the second amino acid in the cytoplasmic region from arginine to cysteine (Arg618Cys). This mutation showed complete association with the Silver phenotype across multiple horse breeds, and was not found among non-Silver horses with one clear exception; a chestnut colored individual that had several Silver offspring when mated to different non-Silver stallions also carried the exon 11 mutation. In total, 64 Silver horses from six breeds and 85 non-Silver horses from 14 breeds were tested for the exon 11 mutation. One additional mutation located in intron 9, only 759 bases from the missense mutation, also showed complete association with the Silver phenotype. However, as one could expect to find several non-causative mutations completely associated with the *Silver *mutation, we argue that the missense mutation is more likely to be causative.

**Conclusion:**

The present study shows that *PMEL17 *causes the Silver coat color in the horse and enable genetic testing for this trait.

## Background

**Figure 1:**
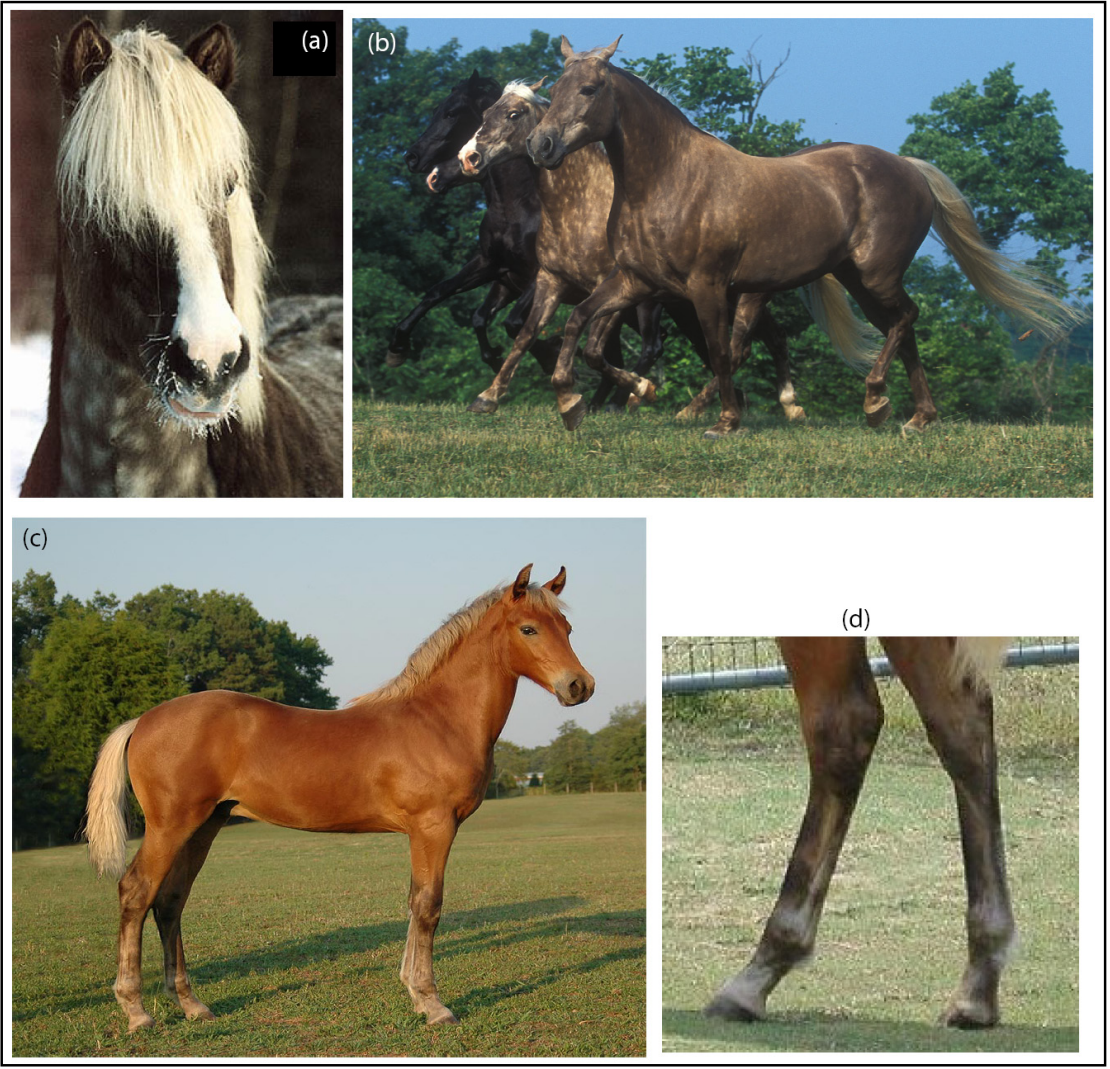
**Phenotypic description of Silver colored horses**. **A. A Black Silver Icelandic horse**. A genetically black horse that exhibits the typical silver phenotype with a dark body with dapples and a shiny white mane and tail. Photo: Tim Kvick. **B. Two Black Silver Rocky Mountain Horses**. Photo: Bob Langrish. **C. A Brown Silver Morgan horse**. A genetically brown horse that shows the silver phenotype with the mane and tail diluted from black to white and the lower legs diluted from black to dark greyish. Photo: Laura Behning. **D. The legs of a Brown Silver horse**. The lower legs are diluted from black to greyish. Photo: Laura Behning.

**Figure 2:**
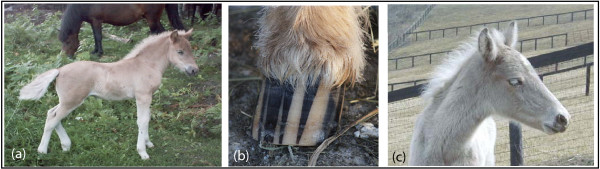
**Phenotypic description of Silver colored foals**. **A. A Silver colored Icelandic horse foal**. Silver foals are generally very pale on the body with white mane and tail. Photo: Elsa Storgärds. **B. A striped hoof of a Silver colored Icelandic horse foal**. Photo: Tim Kvick. **C. White eyelashes of a Silver colored Rocky Mountain Horse colt**. Photo: Unknown.

**Figure 3:**
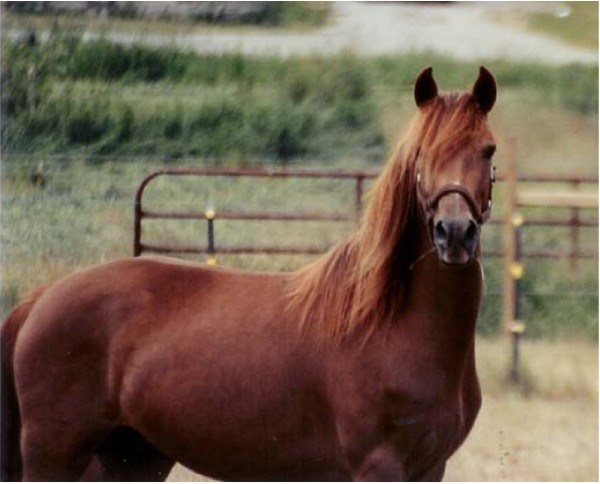
**A chestnut Morgan horse that carry the *Silver *mutation**. This particular individual (Amanda's Suzie Q) indicate that the Silver mutation in horses has little or no effect on pheomelanin (as mane does not seem to be diluted). Photo: Anthony Domire JR.

Hair color clearly plays a critical role in camouflage, social communication, sexual and artificial selection, and as protection against solar radiation [1]. Mammalian hair shafts exhibit a wide range of shades. The shades reflect variation in the production of eumelanin (black) and pheomelanin (red) pigments and give rise to colors that humans perceive as black, red, yellow, gray, or white hair fibers. The Silver (Z) coat color in horses shows an autosomal dominant inheritance and is characterized by dilution of the eumelanin, but with little or no effect on pheomelanin. Interestingly, the effect of the *Silver *mutation is most visible in the long hairs of the mane and tail [2]. A black Silver horse exhibits a phenotype consisting of a slightly diluted body, often with dapples, and a shiny white or flaxen mane and tail (Figure 1A, B). In contrast, the red body color of a bay Silver horse is believed to stay unchanged apart from the legs that change from black to dark grayish, whereas again the long black hairs in the mane and tail are diluted to a mixture of white and gray hairs (Figure 1C, D). Silver colored foals are very pale on the body with white mane and tail (Figure 2A) and often display characteristic features like striped hooves and white eyelashes (Figure 2B, C). Horses that are homozygous (*ZZ*) for *Silver *seem to exhibit a more diluted coat color compared to the heterozygous (*Zz*) horses, but this indication needs to be verified. A chestnut or sorrel horse that only express pheomelanin, will not show any obvious phenotypic effects of the *Silver *mutation (Figure 3), however it is possible that there exist subtle effects of dilution on pheomelanin.

Two of the most important proteins in melanogenesis are the melanocyte-stimulating hormone receptor (MC1R) and the agouti protein [3]. Chestnuts carry a missense mutation (Ser83Phe) in MC1R and only express red pheomelanin [4]. As the Silver coat color can be difficult to identify in young horses, many of these are classified as dark chestnuts or flaxen. One characteristic feature of a Silver horse is that they are often born with striped hooves (Figure 2B, T. Kvick, pers. comm.). These stripes usually disappear after about one year. However, it is still unknown how strong this association is both within and across breeds. Silver is a highly desirable, but not frequent, coat color in certain horse breeds. It is relatively common in the Icelandic horse population, the American Miniature Horse, and the Rocky Mountain Horse. Silver is also present in the Ardenne, the Morgan Horse, the American Paint Horse, the Quarter Horse, the American Saddlebred, the Shetland pony and the Norwegian Nordland, as well as sporadically observed in Welsh ponies, Arabians and Swedish Warmbloods. The Silver phenotype can also be found in other breeds closely related to the above breeds. The *Silver *mutation was possibly present within the Nordic horse breeds before the colonization of Iceland during the 9^th ^century as it is present in the Icelandic horse population on Iceland and import of horses to Iceland was prohibited already during the 10^th ^century.

**Figure 4:**
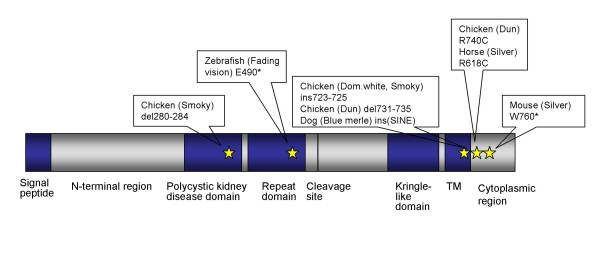
**A schematic picture of the PMEL17 protein with domains and known mutations**. The transmembrane (TM) protein PMEL17 has previously been shown to regulate hypopigmented phenotypes in mouse, chicken, dog, and zebrafish. The location of known mutations associated with hypopigmentation in these species are indicated. R740C in chicken (Dun) is at the same location as the R618C in the horse (Silver).

Mutations in *PMEL17/SILV *have previously been shown to regulate hypopigmented phenotypes in mouse, chicken, zebrafish, and dog [5-9]. *PMEL17 *encodes a transmembrane protein called pre-melanosomal protein 17 or PMEL17 (Figure 4). PMEL17 is involved in the production of eumelanin and is present in the melanosome, but its precise function remains controversial [10]. It might even be so that this protein has an additional role separate from that in melanosome biogenesis. One interesting characteristic feature of PMEL17 that seems necessary for normal melanin production is that it forms non-pathological amyloid fibrils [11,12]. Furthermore, it has been shown that the *PMEL17 *gene is expressed in early cranial melanoblasts in the mouse [13], suggesting an important role during development. The *PMEL17*-mutants identified in different species provide an opportunity to study PMEL17 protein function and its role in the pigmentation process.

The silver *(si) *mutation in mice consists of a point mutation that leads to a premature stop codon and a truncated protein missing the last 25 amino acids [6], although it was first reported that the silver mutation in mice consists of a single base insertion that leads to a frameshift and an elongation of 12 residues of the protein [5]. The effect of the mutation results in premature graying of the hair due to loss of follicular melanocytes [14]. In frame insertion/deletions in the same gene are associated with the Dominant white, Dun and Smoky coat colors in the chicken [7]. The zebrafish mutant *fading vision (fdv) *exhibit defects in vision and hypopigmentation and has a point mutation in *PMEL17 *leading to a truncated protein [8]. The merle patterning of the domestic dog is characterized by patches of diluted pigment and is caused by a retrotransposon insertion in the border of intron 10 and exon 11 of *PMEL17 *[9]. Dogs that carry the merle mutation suffer from both auditory and ophthalmologic abnormalities. These defects are similar to those of the human auditory-pigmentation disorder Waardenburg syndrome [9]. Both the dog and the zebrafish mutants show pigmentation defects in both the coat and in retinal pigment epithelium (RPE). No eye defects have been reported for the mouse and chicken mutations. No mutations in human *PMEL17 *associated with variation in pigmentation have yet been described, but they are likely to exist. The predicted phenotype for such mutants could perhaps be red or blond hair color, fair skin and lightly colored eyes. This study describes complete linkage between the *Silver *locus and the *PMEL17 *gene, and a missense mutation completely associated with the Silver coat color.

## Results

### Genotyping and linkage analysis

Markers from a genome scanning panel known to be evenly spread throughout the horse genome were used for linkage mapping of the Silver phenotype [15]. The stallion was heterozygous for 41 out of the total 78 microsatellite markers, protein- and blood group polymorphisms that were tested. The informative markers represented 25 out of the 32 different horse chromosomes. Genotyping was performed within a half-sib family consisting of one stallion with 34 offspring and their 29 non-Silver dams. The markers were analyzed for pair-wise linkage using the TWOPOINT option of the program CRI-MAP [16]. Significant linkage was found between the Silver phenotype and the marker TKY284 (θ = 0, z = 9.0) at ECA6. These data revealed *PMEL17 *as an outstanding candidate gene for the Silver dapple phenotype in horses as *PMEL17 *previously had been mapped by fluorescence in situ hybridization (FISH) to the same chromosomal region (ECA6q23) as TKY284 [17]. Further, mutations in *PMEL17 *in other species seem to show specific dilution or inhibition of the black pigment [10], as seen in the Silver phenotype in horses. Blasting the non-repetitive flanking sequence of the microsatellite marker TKY284 against the human genome sequence did not reveal any hit to the corresponding region on human chromosome 12, hence we could not get a rough idea of its distance from *PMEL17*.

### DNA sequencing of PMEL17

**Figure 5:**
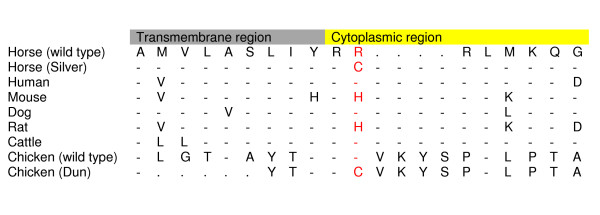
**Amino acid alignment of the end of the transmembrane region and beginning of the cytoplasmic region of the PMEL17**. Amino acid alignment of the end of the transmembrane region and beginning of the cytoplasmic region of the PMEL17. The site of the silver horse mutation is highlighted. Sequence identities are indicated by dashes and insertion/deletion differences are indicated by dots.

The entire *PMEL17 *gene, except parts of the long introns 1 and 3 as well as a repetitive region within intron 6, was sequenced in Silver and non-Silver horses for mutation detection (Accession number: DQ855465). As there was no DNA sequence publicly available from exon 1 to exon 5 of *PMEL17*, this part was obtained by 5' RACE experiments (Accession number: DQ855466). We sequenced 139 bases of intron 1, 128 bases of intron 3 and approximately 1000 bases of intron 6 of which the sequence was disrupted by a repetitive element (mainly a repetition of T) that we were not able to sequence through completely. When the obtained (not complete) sequence of intron 6 was run through RepeatMasker [18] two short interspersed elements (SINEs) were identified. In total, 5.3 kb of *PMEL17 *was sequenced. For almost the entire gene three Silver colored individuals (one homozygote and two heterozygotes) and three non-Silver horses (bay or black) of the Icelandic breed were sequenced. The sequence comparisons revealed two polymorphisms associated with the Silver coat color within the six individuals selected for sequencing. One polymorphism consists of a C (wild type) to T (Silver) transition at the fifth base within exon 11. This is a missense mutation that changes the second amino acid in the cytoplasmic region from an arginine to a cysteine (Arg618Cys). An amino acid alignment of the end of the transmembrane region and beginning of the cytoplasmic region of the PMEL17 that include the missense mutation is shown in Figure 5. See Figure 4 for location of the missense mutation in the PMEL17 protein. The other SNP consists of an A (wild type) to T (Silver) transversion and was identified at nucleotide position 48 in intron 9. The distance between the two SNPs is 759 bases. The two SINEs were present in both Silver and non-Silver horses. For one of the SINEs (143 bases long) we obtained the complete sequence, which did not reveal any variation between Silver and non-Silver horses. However, as the other SINE was not completely sequenced (235 invariant bases was obtained) we could not fully evaluate any potential role in the Silver coat color.

### Association of DNA polymorphisms and the Silver allele

There was a complete association with allele 177 of microsatellite marker TKY284 and the Silver phenotype within all tested horse breeds, except for the Rocky Mountain Horse. Two Rocky Mountain horses were homozygous for the exon 11 missense mutation (T/T) but heterozygous 175/177 at TKY284. One additional Rocky Mountain Horse was heterozygous for the missense mutation (T/C) and heterozygous for TKY284 (169/175).

In order to test if the two identified SNPs were associated with the Silver coat color we genotyped the polymorphisms using pyrosequencing in 14 different horse breeds of which six contained Silver horses (Table 1). Both mutations showed complete association with the Silver phenotype across multiple horse breeds, and also were absent in non-Silver horses from the same breeds with one clear exception; a chestnut colored individual (Figure 3) that had several confirmed Silver offspring when mated to different non-Silver stallions. Hence, these results cannot exclude the intronic mutation from being causative.

**Table 1:** Numbers of individuals from different breeds tested for *PMEL17 *mutations associated with the Silver phenotype in horses.

	**Exon 11**			**Intron 9**		
	Silver		Non Silver	Silver		Non Silver
				
**Breed**	TT	CT	CC	TT	AT	AA
Icelandic horse	1	49	40	1	30	22
American miniature	1	5	8	1	4	6
Rocky mountain horse	2	3	3	2	3	4
Morgan horse		4	3		3	2
Swedish warmblood		2	3		1	3
Ardenne		1	3		1	3
Connemara pony			4			2
Shetland pony			4			4
Haflinger			4			3
Thoroughbred			3			3
Welsh pony			3			3
North Swedish horse			3			2
Norwegian fjordhorse			2			2
New forest pony			2			2

**Total**	**4**	**64**	**85**	**4**	**42**	**61**

## Discussion

The results of the present study strongly indicate that the Silver coat color in horses is caused by a mutation in *PMEL17*. This conclusion is based on the observation of no recombinants between *PMEL17 *and *Silver *in a pedigree material and the identification of a haplotype, composed of sequence variants in intron 9 and exon 11, showing complete concordance with the presence of *Silver *across six different breeds. Furthermore, the specific inhibition of the production of black eumelanin but with no visible effects on red pheomelanin is in perfect agreement with the observed phenotypic effects of previously described *PMEL17 *mutations in mouse and chicken [5-7]. We also describe a candidate causative missense mutation Arg618Cys that is a non-conservative substitution at a conserved site and mutations in the near vicinity cause a similar phenotype in chickens [7]. The second mutation showing a complete concordance with *Silver*, located at position 48 bp in intron 9, cannot be excluded at the present time but it appears less likely as causative since it occurs in an intronic region not well conserved among mammalian species [19]. We find it more likely that this intronic mutation was present on the ancestral haplotype in which the *Silver *mutation occurred and has not yet been separated by recombination events; it is located only 759 bases from the Arg618Cys missense mutation.

The *PMEL17 *mutations identified in other species have a more dramatic effect on the amino acid sequence (Figure 4). A short interspersed element (SINE) insertion at the boundary of intron 10 and exon 11 within the *PMEL17 *gene in dogs is associated with the merle coat color patterning [9]. It was also discovered that deletions within the oligo (dA)-rich tail of the SINE restored normal pigmentation in the dogs. In the mouse two different *silver *mutations have been described, of which the most widely referenced one leads to a premature stop codon and truncation of the protein so that the last 25 amino acids are missing [6]. This mutation also affects the cytoplasmic domain of the Pmel17 protein so that endoplasmatic reticulum (ER) export and endocytic signals are lost [20]. In the zebrafish mutant the hypopigmentation is seen in both the retinal pigment epithelium (RPE) and body melanocytes [8]. Also in this species the mutation leads to a truncation of the PMEL17 protein. This mutation results in a premature stop codon that is located within exon 8. Several different *PMEL17 *mutations associated with inhibition of black pigment have also been documented in the domestic chicken. In this species both insertion and deletion polymorphisms were associated with hypopigmentation [7]. However the exact role and importance of each mutation in diluting the pigment is not clear. Interestingly, the *Dun *allele in chicken carries both a 12 base pairs insertion and the same missense mutation as the horse. This supports our hypothesis that the identified missense mutation may be the causative mutation in horses. It is possible that the introduced cystein residue is enough to disrupt the protein domain in the beginning of the cytoplasmic region, however this remains to be investigated. This region of the PMEL17 protein is a rather well conserved region between species. Of the mammals, the majority has at least two arginines in the beginning of the cytoplasmic region. Also the chicken and other vertebrates have arginines in these positions (Figure 5). Clearly, the missense mutation found in Silver colored horses occurs in a region of the PMEL17 protein that appears critical for proper eumelanin formation. Future studies could, for example, involve analysis of the consequences of the missense mutation for PMEL17 localization and function in cell culture models. Although the identified missense mutation resides in the same region of PMEL17 as the mutations in several other species (Figure 4), it was perhaps a bit surprising that we did not find any additional causative mutations, as the impact on the aminoacid sequence is less drastic in the horse. In line with this future experiments will attempt to completely sequence the longest SINE in intron 6 to fully evaluate any potential role in the Silver phenotype.

The occurrence of *PMEL17 *gene mutations is rare. Considering how well the laboratory mouse is studied it is surprising that only two *PMEL17 *mutations has been identified in this species [5,6]. Several other coat color genes in the mouse carry many more mutations, like for example the transcription factor MITF that carry over 20 different mutations several of which are loss-of-function mutations [21]. There could be several explanations for this rare occurrence of *PMEL17 *gene mutations in different species, of which the most likely seems to be 1) That there actually are existing mutations but they do not have an effect on pigmentation and are therefore missed, 2) That the mutations are lethal. The fact that all of the mutations within the *PMEL17 *gene identified in different species so far – except the zebrafish – are located within or near the last exons implies that mutations in the "earlier" exons could lead to total loss of PMEL17 function and that this perhaps is lethal. This in turn implies that PMEL17 has a function outside melanosome biogenesis as pigmentation is not critical for survival.

One still unanswered question is what the phenotype of a complete loss-of-function is in mammals? As the mutation in the zebrafish creates the most truncated version of the PMEL17 protein identified today, it can possibly shed some light on this question. This mutant is called *fading vision *and lacks the terminal 355 amino acid residues that encodes for one domain and two motives important for localization and function of PMEL17. These include the transmembrane domain, the proteolytic cleavage site and the AP3 binding motif [8]. The mutation seems to result in a total loss of function of the PMEL17 protein in zebrafish as it is shown that the mutation not only has an effect on melanosome biosynthesis, but also is important for normal vision development [8].

Ocular abnormalities caused by a syndrome called Anterior Segment Dysgenesis (ASD) are segregating in the Rocky Mountain Horse breed [22,23]. An unexpected high fraction of the diseased animals in a study of 514 Rocky Mountain horses had the Silver coat color [22]. The clinical and histological signs vary from minimal to quite severe defects in the frontal part of the eye. Interestingly, many of the eye defects observed in the Silver horses are similar to those associated with congenital aniridia or malformation of the anterior segment in humans [23]. The ASD syndrome also has a relatively close resemblance to the defects observed in *Small eye *mice and rats [23]. Microphthalmia is well described in homozygous blue merle Australian Shepherd dogs [24]. It is hypothesized that horses homozygous for the *Silver *mutation have more severe symptoms of the ASD syndrome than heterozygotes [25]. The ASD syndrome is also present in the Kentucky Saddle horse and Mountain Pleasure horse breeds [26], both closely related to the Rocky Mountain Horse. However, in ASD it is the morphology of the eye that is affected and not the pigmentation. Further, in several horse breeds no eye defects have been detected among silver individuals. The ocular defects could therefore be a founder effect. This is in line with the fact that part of the horses examined for ASD could be traced back to one founder animal [23].

The majority – if not all – of the Silver horses in the Icelandic horse breed have striped hooves during the first year of their lives (T. Kvick, pers. comm.). These observations are based on about 100 Silver colored foals and even more non-Silver foals of the Icelandic horse breed. The stripes are vertical and broader at the base, i.e. "triangular" (Figure 2B). It is tempting to speculate that the striped pattern could be associated with a particular color or pattern of the hair on the leg just above the striped hoof in these silver horses. In fact these horses are dappled on the legs right above the hoof (visible when shaving the hair) and this might be related to the stripes. The molecular mechanism for how different types of spatial pattern are formed in animals is largely unknown. However, several studies of the striped pigment pattern in the zebrafish provide basic knowledge to investigate this further [27-31]. The striped pattern in the zebrafish is caused by a specific distribution of three types of pigment cells and recent findings suggest that cell-cell interactions among the pigment cells play a key role for striped pattern formation [28]. It was also found that pigment cells form ordered and layered structures in both striped and interstriped regions that are not seen in non-striped animals [28,29].

## Conclusion

The present study strongly suggests that *PMEL17 *causes the Silver coat color in the horse. Moreover, our results indicate that a missense mutation (Arg618Cys) in *PMEL17 *is likely to be the causative mutation.

The result of this study enables the use of genetic testing to identify horses that carry the *Silver *mutation. A genetic test could be used to distinguish this coat color from other very similar colors. Silver colored horses are likely to be missed and the frequency of Silver horses to be underestimated due to inaccurate identification. As this is a quite popular coat color in certain horse breeds, like for example the Icelandic horse, there is an interest for a genetic test from the horse breeding industry, as breeders would like to know if the animal is homozygous or heterozygous for the mutation or if the horse is a hidden carrier of the mutation, as for example could be the case if the horse is Chestnut or Gray.

## Methods

### Animals

A pedigree consisting of one half-sib family with a *Silver *heterozygous Icelandic horse stallion, 34 of his offspring and 29 of their non-Silver colored dams were used for linkage mapping (some offspring were full-siblings). Seventeen of the offspring had the Silver coat color. For DNA sequencing of the *PMEL17 *gene and polymorphism detection six Icelandic horses were used; one homozygous for *Silver*, two heterozygous for *Silver *and three non-Silver horses. The identified mutation were tested in the following horse breeds; Icelandic horse (including the individuals within the half-sib family), Rocky Mountain Horse, American Miniature Horse, Morgan horse, Ardenne, Swedish Warmblood, Norwegian Fjord, Connemara pony, Thoroughbred, Welsh pony, Shetland pony, New Forest pony, North Swedish horse, and Haflinger (Table 1). All non-Silver horses tested in Table 1 are either black or bay to avoid hidden carriers, or from breeds known not to comprise Silver horses (i.e. Haflinger and Norwegian Fjord; in these two breeds all horses are fixed for coat colors known to be regulated by other loci). DNA and RNA preparations were performed from hair and whole blood according to standard procedures.

### Genetic markers, genotyping and linkage analysis

Microsatellite markers distributed over the horse chromosomes were selected for genotyping from a genome scanning panel [15]. In total, we have typed 41 markers in the half-sib family of which 34 were mirosatellite markers, five were protein polymorphisms (TF, ES, ALB, PGM and A1BG), and two were blood group polymorphisms (A and D system). The microsatellite markers were genotyped essentially as described in [15]. Briefly, PCR reactions were performed as described in [32]. Pairwise linkage between all markers was performed using the TWOPOINT option within the program CRI-MAP [16]. Two-point analysis was used for testing pair wise linkage between all markers.

### Sequencing of PMEL17

Primers for sequencing *PMEL17 *were designed from the published cDNA sequence that contained exon 5 to exon 11 (Accession number: AF076780) [33] and when sequence from introns was achieved new primer pairs were designed. The lacking 5' UTR to exon 5 region of *PMEL17 *gene was sequenced using primers designed from the cDNA sequence obtained in RACE experiments, as well as one horse specific primer in the 5' UTR region designed from a horse specific sequence obtained from the NCBI trace archive database [34]. There was also lacking sequence in the end of exon 11 that was obtained using one conserved primer in the 3' UTR. Long-range PCRs were performed over intron 1 and intron 3 by using the Expand Long PCR kit (Hoffmann-La Roche Ltd, Basel, Switzerland). All primer sequences that were used are listed in Table 2. The primers were designed with Oligo 5.0 (Molecular Biology Insights, Cascade, CO) or Primer 3 (Whitehead Institute for Biomedical Research, Cambridge, MA). PCR was used for amplification of the gene. The amplification was performed in a total volume of 25 μl including 7.5 pmol of each primer, ~30–50 ng of DNA template, 0.2 mM dNTP, 1.9 units AmpliTaq Gold polymerase, 1.5 mM MgCl_2 _and 1 × PCR buffer. The PCR profile consisted of 10 min at 95°C, 5 cycles of 40 s at 94°C, 40 s at 61/57°C and 2 min at 72°C, 35 cycles with 40 s at 94°C, 40 s at 57/55°C and 2 min at 72°C, ending with an extension step for 15 min at 72°C. All PCR products were checked by agarose gel electrophoresis and visualized by EtBr staining before DNA sequencing. The PCR-products were sequenced using MegaBace sequencing kit (Amersham Biosciences, Uppsala, Sweden) and loaded onto a MegaBace 1000 capillary instrument (Amersham Biosciences, Uppsala, Sweden). The sequences were analysed using the Sequencher 3.1.1 software (Gene Codes, Ann Arbor, MI).

**Table 2:** Primer sequences for the sequencing of horse *PMEL17*

**Primer sequence**	**Forward primer (5'-3')**	**Reverse primer (5'-3')**
5'UTR-Intron1	GGATCCCTTGTCAGTTTTGC	AGGAGAGGAAAAACCAGAGC
Exon 1-Exon 2 (LR)	GATGGATCCAGTGTCAGAGATG	ACTCTGGATACAGCTGCCTGTT
Intron 1-Exon 2	CGTGGGATGACGTTATCTTCT	AGTCAGGCCCCTGAATTTCT
Exon 2–3	GTCTCAAGGCAGCTCAGGAA	CATTGATGATGGTGTTGTTGG
Exon 3–4 (LR)	CTACACTGGTTGGGGCAAAT	TCTGAGACAGAGGGCCAGAT
Exon 3-Intron 3	GGTGGCCCTGAAGATCAGTA	AGGGAATTGGAGCCCTTAGA
Intron 3-Exon 4	CTCTCTGGGAGCCGTGTTAG	CCCAGGTCTTCCAGACGTAA
Exon 4-Intron 4	TCCCCAGGAACCTGATGATA	CTTCAGAGGTGGGACCAGAG
Exon 4–5	TCCCCAGGAACCTGATGATA	GCGGTGGTAGACAGTCACTT
Exon 5–6	AGTGTCGGGGCTGAGCAT	CAGGCCACAGCTTGTCTTTT
Exon 5–6(2)	TGCCCCTCGCTCACTCCCGCTCAGCCT	CATGAATGGGCTGGCATCTGGA
Exon 6–7	GGTAACGGTACAGAGTTGGTGGAA	GGACGATGTCCAGAGTGAGGGA
Exon 6–7(2)	AGGTGCCAACTGCAGAGC	GGACGATGTCCAGAGTGAGG
Exon 7-Intron 8	ATGGCACAGCCACCTTATTC	GAAAGGTGTCAGTTTAGGTCAGG
Exon 7–10	CCAGAGCCCCCTGCTGGATGG	TATATCAGAGATGCAAGCACCATA
Exon 9-Intron 10	AATGTGTCTTTGGCTGATGC	TCTGCCCCTCTTACAGGTGA
Intron 10-Exon 11	GCAGGGAAGCTTGTAGAGTGA	CTCTCACCAAAGGGGGAAG
Exon 10–11	AGAGGCAGGCCTTGGGCAG	TGCTCTCACCAAAGGGGGAAG
Exon 11–3'UTR	CAGGCGCAGACTTATGAAGC	AGGGAAGWCTGSRGRAAABA
Pyroseq. PCR primers, Ex 11	Biotin-TCCATTGCTTACCAGTTTCCTT	CTCACCAAAGGGGGAAGAG
Pyroseq. Seq primer, Ex 11	-	GCCCTGCTTCATAAGTCTG
Pyroseq. PCR primers, Int.9	CATGCCTGGTAGGTACTTGGA	Biotin-CCTCTTGACCTGTGAGCAGA
Pyroseq. Seq primer, Int 9.	GGGGAGTGGGCAGAGGCT	-
RACE primer	-	CCGGAGGGCAAAGGTCAGAGGTTG

### 5' Rapid amplification of cDNA ends (RACE)

The 5' end cDNA sequence of the *PMEL17 *gene was obtained by performing a 5' RACE from 1 ng total RNA from skin and melanoma samples using the GeneRacer Advanced RACE Kit (Invitrogen Corporation, Carlsbad, CA) according to the instructions. The 5' end of the gene was amplified using PCR with one gene specific primer in exon 6 (Table 2) and one RACE-primer. The amplification was performed in a total volume of 25 μl containing 50 ng of cDNA, 0.2 mM of dNTP, 1 units of AmpliTaq Gold polymerase, 2.0 mM MgCl_2 _and 1 × PCR buffer. The amplification included 10 min at 95°C, 5 cycles with 40 s at 94°C, 40 s at 57°C and 2 min at 72°C, followed by 35 cycles with 40 s at 94°C, 40 s at 55°C and 2 min at 72°C, ending with 15 min at 72°C. The PCR products were direct sequenced using MegaBace sequencing kit and electrophoresed with MegaBace 1000 capillary instrument. The sequences were analysed using the Sequencher 3.1.1 software.

### SNP analysis using pyrosequencing

The SNPs were analysed using pyrosequencing and performed essentially as described in [35]. Three primers were designed for each SNP, one biotinylated PCR primer, one un-biotinylated PCR-primer and one sequencing primer flanking the SNP of interest. For primer sequences used see Table 2.

## Authors' contributions

EB carried out almost all of the molecular work in this study including genotyping of microsatellite markers, primer design, PCR and preparing samples for DNA sequencing, SNP genotyping by pyrosequencing, chromosome walk experiments, and construction of the RACE libraries. EB also performed the linkage analysis, all sequence analysis and drafted the methods and results section and the tables and figures for this paper.

LA initiated and mentored the study, as well as gave very valuable input and help to draft the paper.

GC provided the American Miniature Horse and Rocky Mountain Horse samples and provided helpful comments on the manuscript.

KS initiated the study, administered the collection of the family material, and provided data for the protein polymorphisms.

SM helped to advise the study, provided most of the horse samples used, double-checked part of the sequence analysis and drafted the protein figure. SM contributed with genotype information for the StockMarks for Horses^® ^microsatellite markers.

GL advised the study, drafted the paper, performed linkage analysis, DNA preparation and pyrosequencing of the samples from GC, long-range PCRs and RACE experiments.

All authors read and approved the final manuscript.
